# The Quorum Sensing Peptides PhrG, CSP and EDF Promote Angiogenesis and Invasion of Breast Cancer Cells *In Vitro*

**DOI:** 10.1371/journal.pone.0119471

**Published:** 2015-03-17

**Authors:** Bart De Spiegeleer, Frederick Verbeke, Matthias D’Hondt, An Hendrix, Christophe Van De Wiele, Christian Burvenich, Kathelijne Peremans, Olivier De Wever, Marc Bracke, Evelien Wynendaele

**Affiliations:** 1 Drug Quality and Registration (DruQuaR) group, Faculty of Pharmaceutical Sciences, Ghent University, Ghent, Belgium; 2 Department of Radiation Oncology and Experimental Cancer Research, Faculty of Medicine and Health Sciences, Ghent University Hospital, Ghent, Belgium; 3 Department of Radiology and Nuclear Medicine, Faculty of Medicine and Health Sciences, Ghent University Hospital, Ghent, Belgium; 4 Comparative Physiology and Biometrics, Faculty of Veterinary Medicine, Ghent University, Merelbeke, Belgium; 5 Department of Medical Imaging, Medicine and Clinical Biology of Small Animals, Faculty of Veterinary Medicine, Ghent University, Merelbeke, Belgium; University of Nebraska Medical Center, UNITED STATES

## Abstract

The role of the human microbiome on cancer progression remains unclear. Therefore, in this study, we investigated the influence of some quorum sensing peptides, produced by diverse commensal or pathogenic bacteria, on breast cancer cell invasion and thus cancer outcome. Based on microscopy, transcriptome and Chick Chorioallantoic Membrane (CAM) analyses, four peptides (PhrG from *B*. *subtilis*, CSP from *S*. *mitis* and EDF from *E*. *coli*, together with its tripeptide analogue) were found to promote tumour cell invasion and angiogenesis, thereby potentially influencing tumour metastasis. Our results offer not only new insights on the possible role of the microbiome, but also further opportunities in cancer prevention and therapy by competing with these endogenous molecules and/or by modifying people’s life style.

## Introduction

To date, cancer figures among the leading causes of death in human societies worldwide. Next to a person’s genetic factors and carcinogens exposure, the human microbiome recently gathered great attention in tumour development as well [1]. *Helicobacter pylori*, a commensal of the human stomach in almost half of the world’s population, was found to cause gastric adenocarcinoma through the CagA and VacA toxins [2]. Colon cancer on the other hand can be influenced by several gut bacteria, producing reactive oxygen/nitrogen species or bacterial toxins (*e*.*g*. Colibactin from *Escherichia coli*) [3, 4].

The vast majority of bacteria inhabit the human intestine, with as many as 10^12^ cells per gram of the average human faeces. Besides, other habitats like skin, mouth, vagina and mammary gland are occupied as well, next to the occasional presence in the blood (bacteraemia or septicaemia). The breast milk in the mammary gland is a continuous source of commensal, mutualistic and potentially probiotic bacteria to the infant gut, containing *i*.*a*. lactic acid bacteria, *Staphylococcus* and *Streptococcus* species [5]. In contrast to the breast milk, the human breast tissue was originally thought to be sterile. However, given the nutrient rich fatty composition of the female breast, together with the blood and lymphatic vasculature, and the diffuse location of the lobules and ducts, bacteria can spread within the mammary glands, resulting in a breast tissue microbiome. Urbaniak *et al*. indeed found a higher abundance of *Proteobacteria*, *Firmicutes* (specifically the class Bacilli) and *Actinobacteria* in the breast tissue, while no signs or symptoms of infection were assigned [6]. These results, serving as a first indication, are eagerly waiting to be confirmed and further investigated towards its pathophysiological role.

Bacteria communicate with each other by the use of signalling molecules in a cell-density dependent manner, a process called ‘quorum sensing’. The signalling molecules differ between the groups of bacteria: while Gram-negative bacteria predominantly use *N*-acyl homoserine lacton (AHL) molecules, the Gram-positive bacteria communicate by the use of oligopeptides; both Gram-positive and Gram-negative bacteria synthesize autoinducer-2 (AI-2) signalling molecules. The quorum sensing process was found to be activated *in vivo* as both the quorum sensing end products (*e*.*g*. biofilms or toxins) and signalling molecules were detected in different areas of the human body. Bacterial biofilms that can be produced after quorum sensing pathway activation can be detected at diverse physiological sites: *e*.*g*. dental plaques produced by *Streptococcus mitis* or *Lactobacillus* species, as well as nosocomial infections with vascular grafts or urinary catheters after biofilm formation by Staphylococci or *Escherichia coli*, were observed [7]. Moreover, *N*-acyl homoserine lacton signalling molecules were detected in human biological samples as well, including sputum, faeces and saliva [8, 9]. Although thus not yet investigated, it is very likely that also quorum sensing peptides are found in the human body, at their site of origin or distributed throughout the human body via the blood circulation.

Despite the increasing evidence of the human microbiome influencing health, up till now, no research has been performed describing the role of quorum sensing peptides on tumour progression. However, important parallels are seen between the bacterial quorum sensing mechanisms and the biochemical and biological mechanisms metastatic cancer cells use to function as a society of malignant cancer cells. The process of biofilm formation has many of the characteristics of metastatic colonization, including motility of cells towards appropriate surfaces, attachment and interaction of cells with each other, surface adhesion and colonization, formation of a complex, heterogeneity,… [10]. Seen these correlations, we hypothesized that cancer cell behaviour can be influenced as well by the quorum sensing signalling peptides, produced by the bacteria of the microbiome. The increasing incidence of breast cancer in women in both developed and developing countries [11], together with the very recent identification of different Gram-positive bacteria in the breast tissue [6], excited the research in the influence of quorum sensing peptides on breast cancer cell behaviour. To this end, we investigated their effect on the human epithelial breast cancer cell line MCF-7/AZ, in order to explain (part) of the microbiome-cancer relationship.

## Materials and Methods

### Cell culture

The epithelial breast adenocarcinoma cell line MCF-7/AZ (ATCC CCL-224) was grown in high-glucose Dulbecco’s Modified Eagle’s Medium (DMEM), supplemented with 10% (V/V) foetal bovine serum (FBS), 1% (w/V) L-glutamine, 100 U/ml penicillin and 100 μg/ml streptomycin (all from Invitrogen/GIBCO, Gent, Belgium) in a humidified atmosphere of 10% CO_2_ at 37°C.

Caco-2 cells, originating from a human colorectal carcinoma, were cultured in high-glucose Dulbecco’s Modified Eagle’s Medium (DMEM) with L-glutamine, supplemented with 10% (V/V) fetal bovine serum (FBS), 1% of nonessential amino acids (100x), 100 U/ml penicillin and 100 μg/ml streptomycin (all from Invitrogen/GIBCO, Gent, Belgium) in a humidified atmosphere of 10% CO_2_ at 37°C.

### Collagen type I invasion assay

Morphology changes of MCF-7/AZ cells were investigated using previously described methods [12]. In brief, 10 000 cells were seeded per well in a 48-well plate, containing 150 μl of collagen type I gel per well, thereby investigating cell morphology 24 hours post-treatment (Leica DMI3000B phase contrast microscope). Peptide solutions (S1 Table) (> 80% purity; all from GL Biochem, Shanghai, China) were prepared using ultrapure water and DMSO, obtaining final peptide concentrations of 1 μM (maximum 0.05% DMSO), 100 nM and 10 nM after 1:10 dilution using growth medium. The placebo sample solely contained ultrapure water; the low DMSO concentration did not influence cell behaviour. Two independent morphology ‘scorings’ were obtained for each of the 3 replicates; peptides were found positive if collagen-invasion or cell-stretching at minimum 2 consecutive concentrations was established. To quantify these visual results, the number of cells containing invasive extensions were counted and compared to the total number of cells in the field.

### Human transcriptome array

Cells treated with either EDF, PhrG or CSP were analyzed in duplicate (independent treatment and analysis) for whole transcriptome expression using Affymetrix GeneChip Human Transcriptome Array 2.0. by AROS Applied Biotechnology A/S (Aarhus, Denmark); RNA expression was compared with placebo treated samples. Data analysis was performed using Transcriptome Analysis Console (Affymetrix) and MetaCore (Thompson Reuters) software programs. The microarray data are deposited at AROS Applied Biotechnology under project numbers A2224 and A2080. The microarray data were submitted to ArrayExpress and received accession number E-MTAB-3191.

### Chick Chorioallantoic Membrane (CAM) assay

The Chick Chorioallantoic Membrane (CAM) assay was performed as described by Sys *et al*.: 6 days after (pre-treated, 100 nM) tumour cell transfer to the fertilized eggs, CAM was microscopically scored and histologically examined after H&E staining [13]. For quantification of microscopically observed neovascularisation, an average CAM score was calculated: the number of blood vessels in the 1 mm diameter ring around the 2 mm radius tumour centre was determined [14]. Significant differences were evaluated using the Mann—Whitney U test.

### Proteome assay

Protein expression was investigated using the Human XL Oncology Array kit (R&D Systems, Abingdon, United Kingdom), following the instructions of the supplier. In brief, cell lysates were obtained after 24 hours of incubation with PhrG (100 nM) or placebo (water) and 84 different proteins analysed in duplo using spotted capture antibodies, followed by incubation with detection antibodies and chemiluminescent visualization. Membranes are finally exposed to X-ray films for 1–10 minutes. The mean (blank corrected, n = 2) pixel density is calculated using ImageJ software and compared with placebo treatment. Significant differences were evaluated using the independent samples t-test.

### Caco-2 permeability assay

For transport studies, Caco-2 cells were seeded at a density of 300 000 cells for each Transwell (Corning Costar, New York, USA) membrane insert filter (0.4 μm pore size, 12 mm filter diameter). Cell culture medium was changed every second day until monolayers were formed (day 21–29); monolayer integrity was evaluated using resistance measurements. Transport experiments were performed in both the apical-to-basolateral and basolateral-to-apical direction in Hanks’ balanced salt solution (HBSS), according to Hubatsch *et al*. [15]. Peptide solutions (PhrG, EDF and EDF-analogue, each 10 μM in HBSS) were added to the donor compartment and aliquots taken from the receiving solution after 30, 60, 90 and 120 minutes of incubation. Aliquots of the donor solutions after 120 minutes were taken as well for calculation of the mass balance. Atenolol (50 μM) and propranolol (20 μM) were used as the low- and high-permeability control, respectively [16]. Peptide samples were analyzed using UPLC-ESI/MS in selected ion monitoring (SIM, monoisotopic mass of the peptides) mode for the peptide analyte, with limits of detection of 0.56–0.58 pmol/ml. The system consisted of a Waters Acquity H-Class Bio-samples FTN (flow rate set at 0.5 ml/min), Waters Acquity H-Class BioQuaternary Solvent Manager, Waters Acquity H-Class column module (set at 30°C) and Waters Xevo TQ-S, equipped with Waters Empower Pro software version 2 and MassLynx version 4.1 (Waters, Zellik, Belgium). Mixtures of water (0.1% formic acid m/V) and acetonitrile (0.1% formic acid m/V) were used to create appropriate gradients (95% to 80% of water in 3 minutes) and ACQUITY UPLC BEH300 C18 column (1.7 μm, 2.1 x 100 mm dimensions) for analysing the peptide solutions (5 μl injection volume). PepT1 transport (apical-to-basolateral direction) was investigated using the dipeptide LY (10 μM) substrate in combination with the quorum sensing peptide.

The apparent permeability coefficient (P_app_ in cm/s) was determined from the amount of compound transported per time and was calculated as follows (sink conditions): P_app_ = (dQ/dt) / (A x C_0_), where dQ/dt is the steady-state flux (pmol/s), A is the surface area of the filter (cm^2^) and C_0_ is the initial concentration of analyte in the donor chamber (pmol/ml).

### Stability in cell medium and human plasma

*In vitro* chemical and metabolic stability was determined in cell medium and human plasma, respectively, using previously described procedures [17]. In brief, 100 μg of peptide was incubated in Krebs-Henseleit buffer pH 7.4 with cell medium/plasma (500 μl) at 37°C while shaking. At predetermined time intervals (*i*.*e*. 0, 6 and 24 hours for cell medium; 0, 30 and 120 minutes for plasma), aliquots were immediately transferred into microtubes containing 1:1 volume of 1% (V/V) trifluoroacetic acid solution in water. The enzyme reaction was further stopped by heating the solution at 95°C for 5 minutes. Next, the samples were centrifuged to precipitate the denatured proteins and the supernatant analyzed using UPLC-PDA. Appropriate placebo solutions were similarly prepared. Assuming first-order kinetics, the rate constant k was obtained from ln(P_t_/P_t0_) = -kt, from which the half-life was determined as t_1/2_ = ln(2)/k.

### Haemolysis assay

A 10 μM stock solution is prepared for each quorum sensing peptide using water, with or without DMSO for solubility reasons. 40 μl of this stock solution is then diluted with 280 μl of 100 mM sodium phosphate buffer, pH 7.4 to obtain the peptide sample. To prepare the Red Blood Cell (RBC) solution, freshly obtained human blood was centrifuged at 1500 rpm for 15 minutes and the precipitate washed three times using freshly prepared 150 mM NaCl solution. After the final centrifugation step, the precipitate was resuspended into 100 mM sodium phosphate buffer pH 7.4 and mixed by inversion. The RBC solution was then diluted 1 to 10 with the same solvent to obtain the diluted RBC solution. To examine the haemolytic properties of the quorum sensing peptides, 320 μl of the peptide sample was mixed with 80 μl of the diluted RBC solution to obtain a final volume of 400 μl and a final peptide concentration of 1 μM. Samples were incubated for 1 hour at 37°C while gently shaking. 100 mM sodium phosphate buffer pH 7.4 and 1% (V/V) Triton X-100 were chosen as the negative and positive control solutions, respectively. Blank solution was obtained by mixing 40 μl of water or aqueous DMSO (60%) with 280 μl of 100 mM sodium phosphate buffer pH 7.4 and 80 μl of diluted RBC solution. After 1 hour of incubation, all samples were centrifuged at 20 000 g for 5 minutes. The absorbance of the supernatant was measured at 405 nm and corrected for the blank absorbance. The percent haemolysis was then calculated using the following equation: [(A_sample_—A_blank_) / (A_positive control_—A_blank_)] × 100

## Results

**Fig 1:**
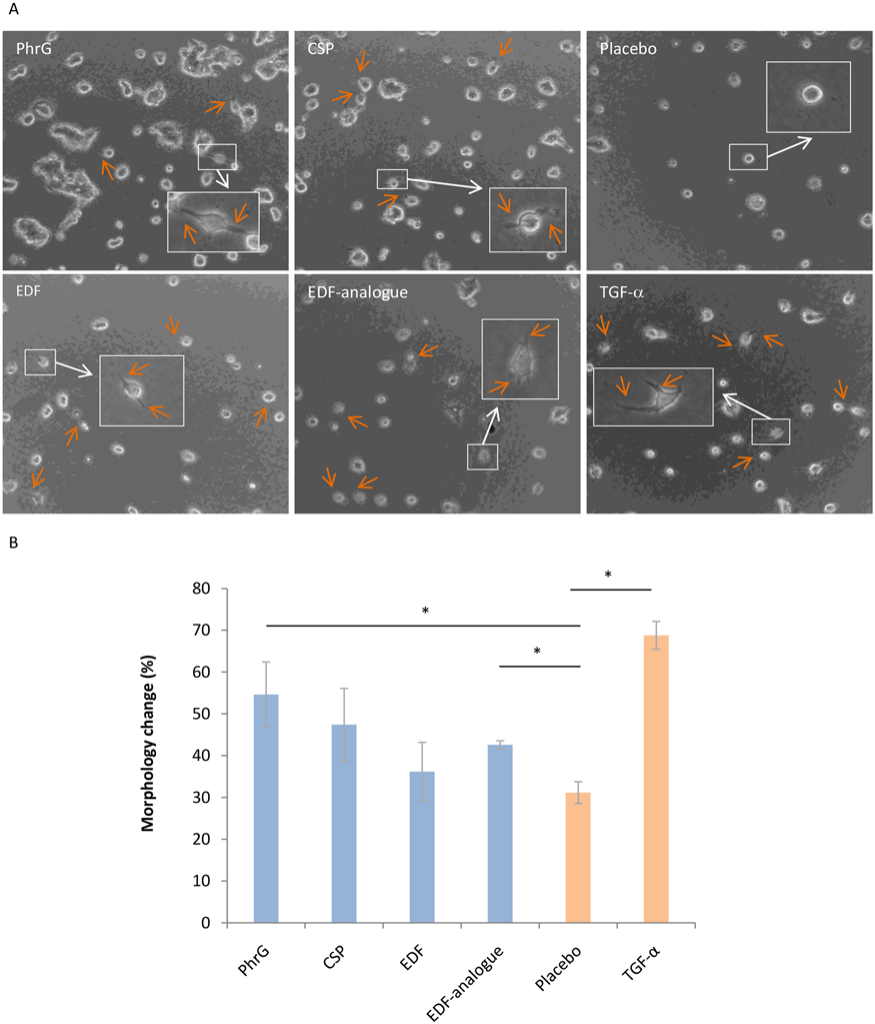
Morphologic changes of non-invasive epithelial MCF-7/AZ cells, induced by quorum sensing peptides at 10 nM. (A) The invasive cellular extensions, observed 24 hours post-treatment with PhrG (EKMIG), CSP (EMRKSNNNFFHFLRRI), EDF (NNWNN) and the EDF-analogue (NWN), indicate tumour-promoting properties of these quorum sensing peptides. The placebo sample serves as the negative control and TGF-α (0.1 μg/ml) as the positive control. (B) Mean (n = 3) number of cancer cells with induced morphology changes: a significant difference is observed between the PhrG peptide or EDF-analogue and the placebo treatment (* p < 0.05, Mann-Whitney U test). Error bars represent SEM values.

To investigate the crosstalk between the quorum sensing peptides produced by the host bacteria and human breast cancer cells, we selected different signalling peptides from the Quorumpeps database (S1 Table) [18]. Four quorum sensing peptides or analogues thereof were consistently (and concentration-dependently) found to induce tumour cell invasion through a type I collagen extracellular matrix: PhrG from *Bacillus subtilis* (EKMIG), a Competence Stimulating Peptide (CSP) from *Streptococcus mitis* (EMRKSNNNFFHFLRRI) and Extracellular Death Factor (EDF) from *Escherichia coli* (NNWNN), together with its analogue NWN (Fig. 1). Other peptides (*e*.*g*. PhrA (ARNQT) from *Bacillus subtilis* or CSP (ESRLPKIRFDFIFPRKK) from *Streptococcus mitis*) did not induce invasive characteristics. These biologically active peptides were found to be sufficiently stable in cell medium (Table 1), meaning that the effects, observed after 24 hours of treatment, can be associated with the original peptide structures. These observations of invasive cellular extensions are linked to an induced epithelial-to-mesenchymal transition (EMT), an important process in breast cancer progression and metastasis and the primary cause of breast cancer-related deaths [19, 20].

**Table 1:** *In vitro* cell medium half-life values of quorum sensing peptides.

Peptide	Sequence	Cell medium half-life (hours)
PhrG	EKMIG	160^a^
CSP	EMRKSNNNFFHFLRRI	45.84
EDF	NNWNN	16.19
EDF-analogue	NWN	42.03

The investigated quorum sensing peptides were found highly stable in cell medium.

^a^ The calculated half-life is 157.89 hours

Up-regulation of the Histone cluster 1 H4 gene (*HIST1H4A-F/H-L*) after EDF treatment, which was observed by our transcriptome expression results (Table 2), assigns tumour progressive characteristics to this quorum sensing peptide. Through its link with β-arrestin 1, EDF induces angiogenesis, thereby promoting the survival of breast cancer cells. Moreover, it promotes cytoskeleton reorganization of breast cancer cells as well through the cofilin pathway, which is crucial for tumour migration [21–23]; these morphologic alterations were already observed in our type I collagen invasion assay. With Notch1 over-expression, a poor clinical outcome of breast cancer is correlated, again promoting angiogenesis and thus tumour progression [24]. Over-expression of the *MTRNR2L2* and *MTRNR2L6* genes, leading to increased synthesis of the Humanin-like proteins 2 and 6 respectively, can be linked to an anti-apoptotic function of the EDF peptide [25]. Finally, up-regulation of *EYA3-IT1* can be associated with increased tumour size and metastasis, due to the tyrosine phosphatase activity of the transcribed proteins, promoting the motility and invasiveness of cancer cells [26].

**Table 2:** Transcriptome alterations after EDF, PhrG or CSP quorum sensing peptide addition to MCF-7/AZ cells.

Gene symbol	Fold change	ANOVA p-value	Description
**Upregulated**			
*MIR548W*	1.92	0.566	MicroRNA 548w
*HIST1H4A-F/H-L*	1.98	0.330	Histone cluster 1, H4(a-f/h-l)
*MTRNR2L2*	1.69	0.010	MT-RNR2-like 2
*MTRNR2L6*	1.58	0.243	MT-RNR2-like 6
*RNU2–4P*	1.59	0.011	RNA, U2 small nuclear 4, pseudogene
*RNU4–4P*	1.54	0.065	RNA, U4 small nuclear 4, pseudogene
*MIR320C2*	1.67	0.076	MicroRNA 320c-2
*EYA3-IT1*	1.75	0.087	EYA3 intronic transcript 1
*IGLJ4*	1.58	0.011	Immunoglobulin Lambda Joining 4
*SNORD92*	1.58	0.261	Small Nucleolar RNA, C/D Box 92
*FOXP1-IT1*	1.63	0.390	FOXP1 Intronic Transcript 1
*MIR635*	1.65	0.024	MicroRNA 635
**Downregulated**			
*RN5S217*	-1.88	0.127	RNA, 5S ribosomal 217
*FOXQ1*	-1.72	0.338	Forkhead Box Q1
*MAGEB5*	-1.75	0.199	Melanoma Antigen Family B, 5
*MIR4718*	-1.64	0.401	MicroRNA 4718
*LOC100128593*	-1.56	0.084	Uncategorized LOC100128593

Gene expression 24 hours post-treatment, compared to placebo samples. Mean fold change is calculated from duplicate samples (cut off: > 1.5 or < -1.5).

**Fig 2:**
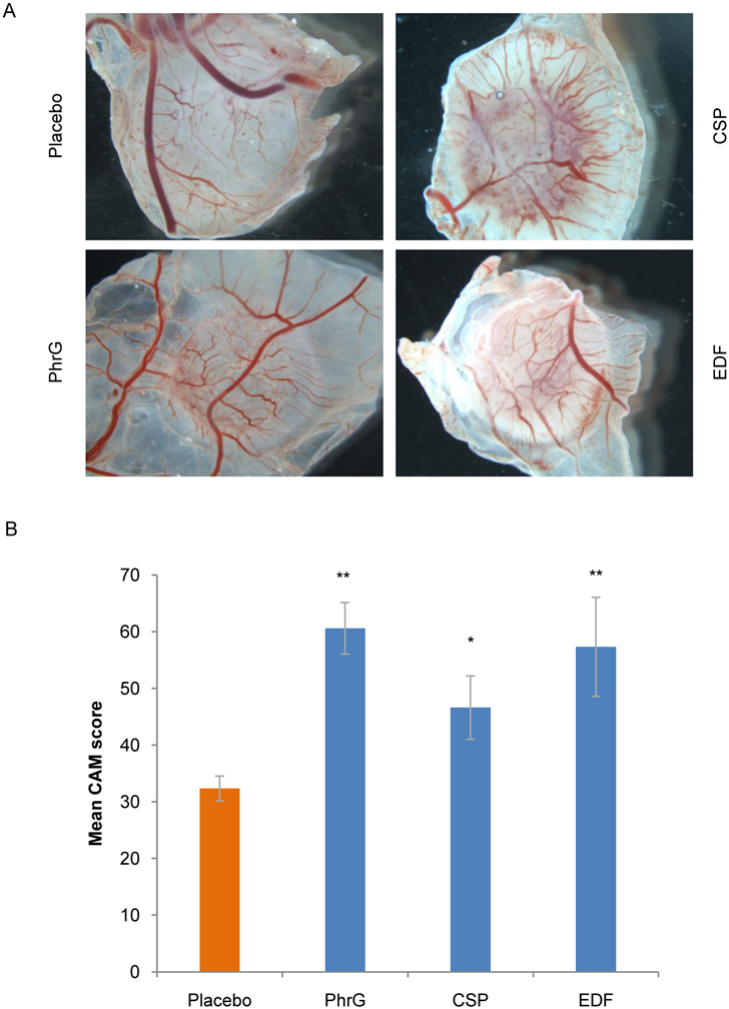
Neovascularisation after quorum sensing peptide treatment (100 nM) of MCF-7/AZ cells on CAM. Placebo sample serves as negative control. (A) Macroscopic images, observed 6 days after (pre-treated, 24 hours) tumour cell transfer to the eggs. (B) Mean CAM score (*i*.*e*. number of blood vessels in the 1 mm diameter ring around the 2 mm radius centre) in the presence of tumour cells (mean ± SEM, n = 5 (PhrG and CSP), n = 3 (EDF) or n = 9 (placebo)). ** p < 0.05, * p < 0.1 (Mann-Whitney U test)

**Fig 3:**
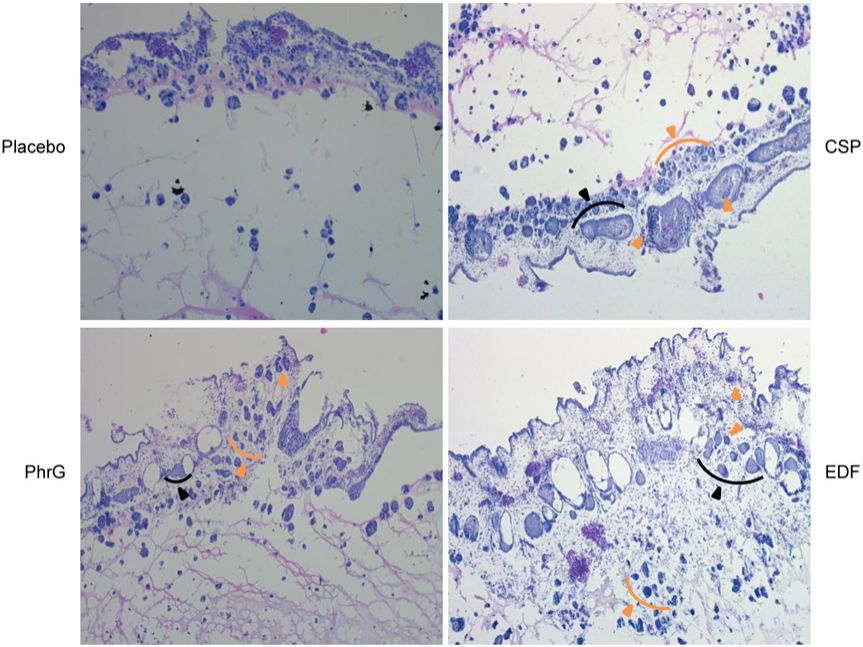
Histological H&E evaluation of the chorioallantoic membrane (CAM). Placebo sample serves as negative control. Tumour cells, when treated with the quorum sensing peptides, moved through the chorionic layer into the mesoderm (orange arrowheads). Clusters of chorionic epithelial cells are indicated by black arrowheads, indicating aggressive tumour cell invasion through the membrane.

The angiogenic properties of the quorum sensing peptides, as postulated by the transcriptome expression results, were confirmed using the Chick Chorioallantoic Membrane (CAM) assay. From Fig. 2, it is clear that all investigated peptides significantly promoted neovascularisation after peptide treatment of the tumour cells, compared to the placebo sample. This recruitment of new blood vessels thereby contributes to the metastasis of tumour cells. Histological evaluation of the membrane confirmed the invasive tumour cell properties through the chorionic layer into the mesoderm, induced by the quorum sensing peptides (Fig. 3). Groups of chorionic epithelial cells thereby indicated an aggressive invasion of MCF-7/AZ cells: the chorion is massively disrupted by the tumour cells, resulting in a collapse of the membrane (black arrowheads in Fig. 3).

**Fig 4:**
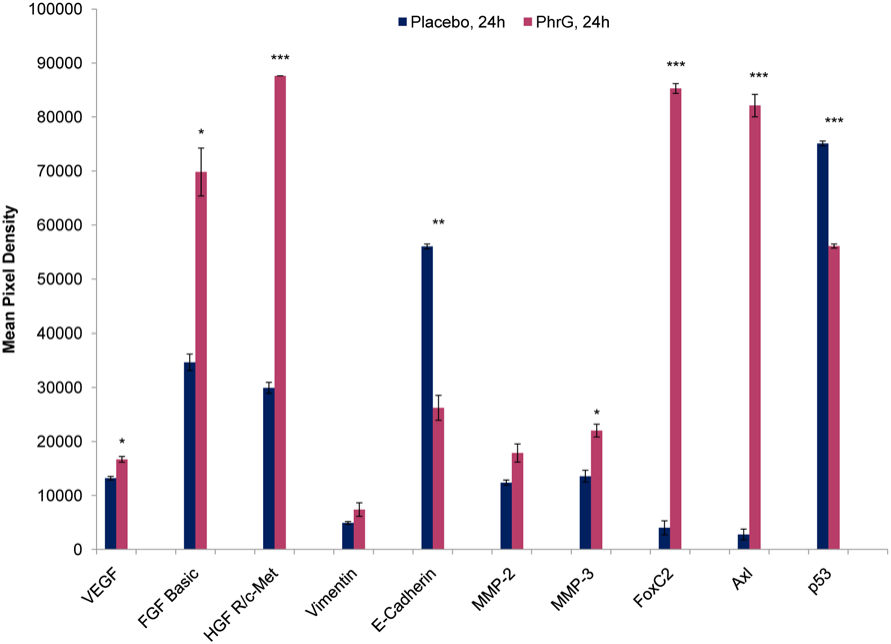
Protein expression after PhrG quorum sensing peptide treatment (100 nM) of the MCF-7/AZ cells. Placebo sample serves as negative control. Mean (n = 2, ± SEM) pixel density of some proteins are given after 24 hours of peptide incubation. * p < 0.05; ** p < 0.01; *** p < 0.001 (independent samples t-test).

The results of quorum sensing peptide induced tumour cell invasion and angiogenesis were confirmed by a protein expression assay (Fig. 4). An increase in Vascular Endothelial Growth Factor (VEGF), Fibroblast Growth Factor (FGF) or Hepatocyte Growth Factor Receptor (c-Met/HGFR), seen after quorum sensing peptide treatment, can be linked to the formation of new blood vessels [27, 28]. Second, the induction of epithelial-to-mesenchymal transition (EMT) of the cancer cells after peptide treatment is validated as well: both the Forkhead box protein C2 (FOXC2) and Tyrosine-protein kinase (Axl) expression are significantly induced [29, 30]; an increase in Vimentin expression, together with a decrease in E-cadherin are associated with EMT as well, leading to tumour cell migration and cancer metastasis [31]. Moreover, a decrease in p53 protein expression can be linked to a reduced activation of apoptosis, together with an increased activation of angiogenesis and tumour growth [32]. The induced expression of C-Met/HGFR is associated with an increase of the tumour growth as well [28].

**Fig 5:**
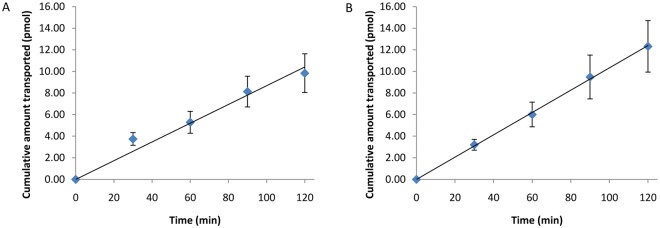
Intestinal permeability of quorum sensing peptide NWN. (A) Mean (n = 5) apical-to-basolateral transport (*i*.*e*. intestinal absorption) of the EDF-analogue (NWN) through a Caco-2 monolayer; (B) mean (n = 6) basolateral-to-apical transport (*i*.*e*. blood efflux) of the EDF analogue. Error bars represent SEM values.

Despite their possible presence in the human breast (cancer) tissue, we investigated the intestinal permeability characteristics of the quorum sensing peptides, produced by commensals of the human gut, as well. Intestinal absorption of the peptides thereby strengthens the effects observed on breast cancer cells. EDF (NNWNN), which is synthesized by the human gut commensal *Escherichia coli*, was found to be impermeable through the human epithelial enterocytes under our experimental Caco-2 conditions (*i*.*e*. P_app_ < 2.9 × 10^-9^ cm/s as LoD = 0.58 nM). However, its tripeptide analogue NWN shows low intestinal permeability kinetics (Fig. 5): a permeability coefficient (P_app,ab_) of 1.41 × 10^-7^ cm/s was calculated for the apical-to-basolateral transport. The efflux ratio (P_app,ba_/P_app,ab_) of 1.10 is doubled using the PepT1 substrate LY: the P_app,ab_ decreases to 6.94 × 10^-8^ cm/s, indicative for the active proton-dependent transport mechanism for peptide absorption. *In vivo—in vitro* correlation studies demonstrated that these *in vitro* P_app_ values correspond with the absorption of about 5–10% in humans (*in vivo*) [15, 33]. Quorum sensing peptide PhrG (EKMIG), produced by *Bacillus subtilis*, only shows very small blood efflux (basolateral-to-apical) transport properties after 120 minutes (1.37 × 10^-8^ cm/s), without significant Caco-2 influx kinetics (LOD = 0.56 nM, so P_app_ < 6.9 × 10^-9^ cm/s).

Once the peptides have reached the blood circulation, they remain sufficiently stable (Table 3), meaning that this pharmacokinetic property will not be the critical parameter for the biological activity observed with these peptides. No haemolytic properties can be assigned to the peptides (1 μM) as well (*i*.*e*. percentage of haemolysis < 1%), after a 1 hour incubation period.

**Table 3:** *In vitro* plasma half-life values of quorum sensing peptides.

Peptide	Sequence	Plasma half-life (hours)
PhrG	EKMIG	>13^a^
CSP	EMRKSNNNFFHFLRRI	8.73
EDF	NNWNN	>13^a^
EDF-analogue	NWN	4.47

The investigated quorum sensing peptides were found sufficiently stable in human plasma, so tumour-promoting properties can be executed at the breast cancer tissue.

^a^ The calculated half-life is 13.16 hours

## Discussion

Our *in vitro* results thus indicate that some quorum sensing peptides promote angiogenesis and induce invasion of human breast cancer cells. Based on these results, different questions related to their biological relevance now arise: (1) Do these quorum sensing peptides reach the breast cancer tissue, (2) Can we inhibit these negative microbiome-related effects, and (3) Does this have consequences for the patient’s life style?

First, very recent investigations have shown that microbial DNA and viable bacterial cells are present in healthy and cancerous breast tissue, thereby indicating that bacteria or their components may influence the local microenvironment. The predominant bacteria in breast tumours were *Escherichia* and *Bacillus* [34]. These findings elicit contradictions with previous insights, where it was assumed that the breast tissue was a sterile location, except for the nipple region; nipple duct contamination with skin flora is generally accepted and plays an important role in breast infection [35]. Clearly, future studies are needed to confirm the presence of a breast tissue microbiome under different personalised conditions and describe the different bacterial species that are present. Next, a thorough exploration of the synthesized quorum sensing peptides in the breast tissue should be performed as well; if found, they can negatively influence the nearby breast cancer tissue.

Quorum sensing peptides, produced by commensal or pathogenic bacteria at distant locations, can reach the breast tissue as well via the blood circulation. *N*-acylhomoserine lacton (AHL) molecules, another group of quorum sensing molecules, are already found in human sputum, faeces and saliva, whereby the last biological sample reflects the systemic AHL concentration [8, 36]. Besides, peptides that are synthesized in the gastrointestinal tract can pass the intestinal barrier as well and consequently reach the blood circulation [37]. Our results indeed indicate that the EDF-analogue NWN, with EDF being synthesized by the gut bacterium *E*. *coli*, can pass the intestinal barrier, thereby reaching the blood circulation. The PhrG peptide of *Bacillus subtilis*, also a gut bacterium, does not show any absorption from the intestinal lumen. Intestinal permeability is however not limited to these small peptide structures: previous studies have indicated that larger peptides and proteins can also cross the normal intestine in an intact form (*e*.*g*. insulin, β-casein) [38–40]. Moreover, the use of permeability enhancers (*e*.*g*. bile salts, fatty acids or glycerides) can increase the intestinal absorption of the quorum sensing peptides as well [41]. Quorum sensing peptides can also enter the circulation through the skin or oral mucosa, seen the highly present bacterial population at these sites of the human body [42] and the skin and mouth mucosa permeability-data already described for peptides [43, 44]. In general, the oral mucosa is considered intermediately permeable, situated between the epidermis and the intestinal mucosa [45]. CSP, the quorum sensing peptide synthesized by the oral commensal bacteria *Streptococcus mitis*, thus possibly can reach the blood circulation after passing the oral mucosa; the same can be observed with the quorum sensing peptide PhrG of *Bacillus subtilis* after passing the skin. This should however be investigated by *in vitro* or *in vivo* permeability assays. Occasionally, bacteria themselves can also be found in the blood circulation: *Bacillus subtilis* and *Streptococcus mitis*, commensals of the gastrointestinal tract or skin and oropharynx, respectively, were found to be present in the blood of cancer patients [6, 46, 47]. PhrG and CSP, when present in the blood circulation, thus can affect the clinical outcome of breast cancer, seen its pro-invasive characteristics on breast cancer cells and angiogenesis-promoting properties. Although thus not yet confirmed, quorum sensing peptides might be available from the blood and influence breast cancer progression.

To answer the second and third question, *i*.*e*. ‘Can we inhibit these microbiome-related effects and does this have consequences for the patient?’, the *in vivo* functionality of the quorum sensing peptides should be investigated to confirm our obtained *in vitro* results. If the quorum sensing peptides are found to be present *in vivo*, our preliminary *in vitro* findings of pro-metastatic effects of quorum sensing peptides can open up new perspectives. First, after identification of the tumour target, quorum sensing peptide-based antagonists can be developed to compete with these endogenous molecules for receptor binding. Tumour progression, induced by the quorum sensing peptide, will then be blocked, indicating therapeutic properties for these antagonists. Second, a person’s life style may be adapted to possibly influence the observed bacteria-related effects. As a change in diet can drastically adapt the microbiota composition, and thus the quorum sensing peptide profile, this may possibly have consequences on cancer outcome [48]. Last, inadequate hygienic measures during *e*.*g*. surgery can induce breast infection as well. Moreover, during lactation, mastitis should be treated carefully to avoid unwanted bacterial infection of the breast tissue as well [49].

The results of this study thus indicate the existence of a crosstalk system between bacteria and breast cancer cells through quorum sensing peptides. More specifically, we showed that these quorum sensing peptides promote breast cancer cell invasion and angiogenesis, and thus that bacteria can potentially influence tumour metastasis. Further research concerning their biological availability should be performed to strengthen these functionality results.

## Supporting Information

S1 Table List of quorum sensing peptides, repeatedly (n = 3) investigated for their effect on tumour cell invasion (Collagen Type 1 invasion assay). (DOCX)
