# Supplementary material for: The Quorum Sensing Peptides PhrG, CSP and EDF Promote Angiogenesis and Invasion of Breast Cancer Cells In Vitro
Source: PLoS One. 2015 Mar 17;10(3):e0119471. doi: 10.1371/journal.pone.0119471 (PMC4363635; doi:10.1371/journal.pone.0119471)
Supplement: S1 Table — (DOCX) [file pone.0119471.s001.docx]

| **Quorumpeps ID** | **Sequence** | **Origin** |
| --- | --- | --- |
| 13 | AIFILAS | *Enterococcus faecalis* |
| 16 | AKTVQ | *Bacillus anthracis* |
| 17 | ALILTLVS | *Enterococcus faecalis* |
| 18 | ARNQT | *Bacillus subtilis* |
| 19 | NNWNN | *Escherichia coli* |
| 30 | DLRGVPNPWGWIFGR | *Streptococcus sanguis* |
| 44 | EKMIG | *Bacillus subtilis* |
| 46 | EMRKSNNNFFHFLRRI | *Streptococcus mitis* |
| 54 | ESRLPKIRFDFIFPRKK | *Streptococcus mitis* |
| 76 | SNLVECVFSLFKKCN | Derived from *Enterococcus faecium* |
| 101 | GLWEDILYSLNIIKHNNTKGLHHPIQL | *Streptococcus pneumoniae* |
| 102 | GLWEDLLYNINRYAHYIT | *Streptococcus pneumoniae* |
| 133 | LFVVTLVG | *Enterococcus faecalis* |
| 151 | NNGNN | Derived from *Escherichia coli* |
| 155 | NWN | Derived from *Escherichia coli* |
| 180 | SGSLSTFFRLFNRSQTQALGK | Derived from *Streptococcus mutans* |
